# The Tool Life and Coating-Substrate Adhesion of AlCrSiN-Coated Carbide Cutting Tools Prepared by LARC with Respect to the Edge Preparation and Surface Finishing

**DOI:** 10.3390/mi11020166

**Published:** 2020-02-05

**Authors:** Tomáš Vopát, Martin Sahul, Marián Haršáni, Ondřej Vortel, Tomáš Zlámal

**Affiliations:** 1 Faculty of Materials Science and Technology in Trnava, Institute of Production Technologies, Slovak University of Technology in Bratislava, Jána Bottu 25, 917 24 Trnava, Slovakia; 2 Faculty of Materials Science and Technology in Trnava, Institute of Material Science, Slovak University of Technology in Bratislava, Jána Bottu 25, 917 24 Trnava, Slovakia; 3 Faculty of Mechanical Engineering, Department of Machining, Assembly and Engineering Metrology, Technical Univesity of Ostrava, 17. listopadu 15, 708 33 Ostrava-Poruba, Czech Republic; 4 Faculty of Mechanical Engineering, Department of Machining Technology, Technická 2, Brno University of Technology, Královo Pole, 616 69 Brno, Czech Republic

**Keywords:** tool life, tool wear, edge preparation, cutting edge radius, surface finish, nanocomposite hard coating, coating-substrate adhesion, brushing, wet microblasting

## Abstract

Nanocomposite AlCrSiN hard coatings were deposited on the cemented carbide substrates with a negative substrate bias voltage within the range of −80 to −120 V using the cathodic arc evaporation system. The effect of variation in the bias voltage on the coating-substrate adhesion and nanohardness was investigated. It was clear that if bias voltage increased, nanohardness increased in the range from −80 V to −120 V. The coating deposited at the bias voltage of −120 V had the highest nanohardness (37.7 ± 1.5 GPa). The samples were prepared by brushing and wet microblasting to finish a surface and prepare the required cutting edge radii for the tool life cutting tests and the coating adhesion observation. The indents after the static Mercedes indentation test were studied by scanning the electron microscope to evaluate the coating-substrate adhesion. The longer time of edge preparation with surface finishing led to a slight deterioration in the adhesion strength of the coating to the substrate. The tool wear of cemented carbide turning inserts was studied on the turning centre during the tool life cutting test. The tested workpiece material was austenitic stainless steel. The cemented carbide turning inserts with larger cutting edge radius were worn out faster during the machining. Meanwhile, the tool life increased when the cutting edge radius was smaller.

## 1. Introduction

The coating-substrate adhesion strength, the hardness of coated cutting tools, and the cleaning of the substrate before deposition are critical factors for producing cutting tools with excellent performence [1].

Xiao et al. investigated the adhesion strength and failure mechanism of the hard CrN coatings deposited on different substrates [2]. The cleaning process of substrates and further plasma pre-treatment of the substrate surfaces are necessary for achieving excellent coating-substrate adhesion [3]. The coating adhesion is strongly affected by the process of plasma pre-treatment [4,5]. Research of Grančič et al. [6] examined the nanohardness and elastic modulus of Ti–B–Si coatings. Furthermore, the high-temperature oxidation resistance was determined. Research of Hudec et al. [7] evaluated the coating-substrate adhesion by a scratch test using a diamond Rockwell tip. The higher content of nitrogen in the films increases hardness but worsens adhesion. The coating-substrate adhesion is affected by the deposition parameters and the coating thickness [8].

Research of Chen et al. [9] showed techniques and approaches used in the measurement of coating adhesion and bond strengths in the coating-substrate systems.

The studies a lot of authors stated the formation of the nanocomposite structure [10,11,12,13,14,15]. The influence of the deposition parameters on the mechanical properties of CrAlSiN-thin coatings was discussed in research of Ding [11] and Kim [16]. Elkaseer et al. [17] presented the results of the FEM modeling of chip formation and surface generation during the turning of stainless steel material. The cutting edge radius size had an essential role in the cutting process. The minimum surface roughness parameters were reached when the feed rate was in a particular range of the original cutting edge radius.

The edge preparation had a significant effect on the cutting forces [18]. The influence of different cutting edge microgeometry (rounding and chamfer) on the quantitative measurement of end burr and chip segmentation in the machining of AA2024-T351 was presented in research of Asad [19]. The effect of the tool material and edge preparation had the greatest percentage contribution to flank wear [20]. Qi Shen et al. [21] investigated the effect of cutting edge microgeometry on residual stresses by FEM. The results from the numerical experiment stated that an increase in the size of the cutting edge radius caused enhanced values of the surface tensile residual stress.

Studies of Bouzakis et al. [22], Uhlmann et al. [23] and Denkena et al. [24] deal with investigation of the optimal form of cutting edge microgeometry. The tested cutting tools were honed by several methods of edge preparation with surface finishing. Denkena et al. [25] described the new way of multi-axis brushing for producing the cutting edge with asymmetrical microgeometry. Biermann et al. [26] formed the asymmetric microgeometry using the method of wet microblasting with a robot for motion control to form the shape and micro size of the cutting edge.

It was necessary to introduce new parameters for an accurate description of the asymmetric cutting edge because the size of the cutting edge radius was not enough. Therefore, Denkena et al. [27] proposed characterization of edge microgeometry by new parameters, *Sα*, *Sγ*, *Δr,* and *φ.* Meanwhile, the approaches of authors as Rodriguez [28] and Wyen et al. [29] used different ways to characterize the shape and size of the asymmetric cutting edge microgeometry.

In research of Lee et al. [30], a new online cutting edge radius estimator for micromachining was determined in order to foresee the cutting edge radius by identifying the drop in the chip production rate. Bai et al. [31] evaluated the surface roughness after milling, with respect to various cutting edge radius sizes.

The new unconventional methods for the cutting edge rounding were discussed in research of authors as Yussefian et al. [32], Karpuschewski et al. [33], Aurich et al. [34], Uhlmann et al. [35] and Bouzakis et al. [36]. Interesting results were attained by Vopat et al. [37], where authors demonstrated a novel method of edge preparation with surface treatment by plasma discharges in an electrolyte.

In this paper, the influence of coating deposition parameters and surface finishing methods on coating-substrate adhesion was determined. Moreover, the effect of the cutting edge radius size on the tool life during turning of the austenitic stainless steel material was investigated.

## 2. Preliminary Experiment

The preliminary experiment aimed to determine the optimal coating deposition parameters for the cutting tools made of cemented carbide. Cemented carbides discs with a diameter of 12 mm were used as samples. The chemical composition was the following: 89.6% of WC (tungsten carbide), 10% of Co (cobalt), and 0.4% of other carbides. This kind of substrate was selected with respect to the PVD (physical vapor deposition) coating process, workpiece material, and semi-finishing strategy in the tool life cutting test. The evaluated results from the preliminary experiment set conditions for the coating deposition for the experiment are in the next section.

### 2.1. Coating Deposition Conditions

**Figure 1:**
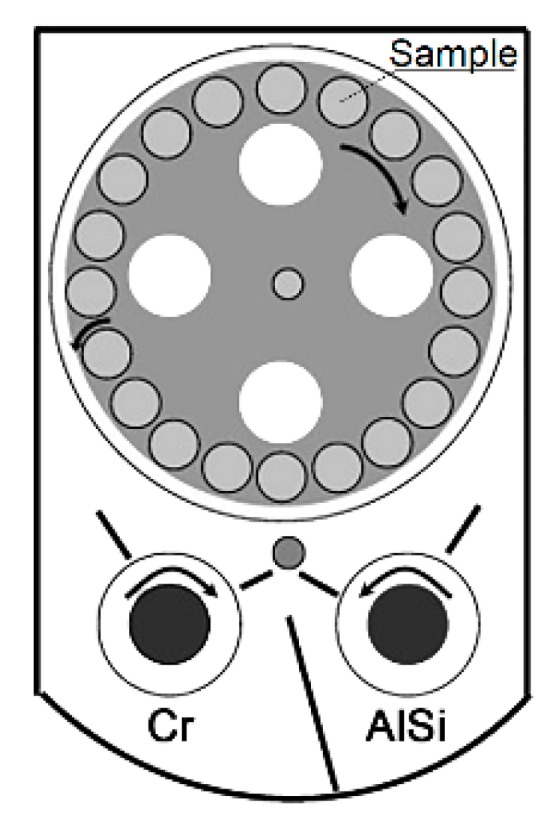
The scheme of coating technique.

An industrial-scale cathodic arc evaporation system with the LARC^®^ technology PLATIT π^80^ + DLC (Diamond-like carbon) was used for coating deposition. Two cylindrical and rotating cathodes were used, and alloyed AlSi (88:12%) and pure Cr (99.99%) were utilized. The coatings consisted of a 200 nm-thick adhesion CrN layer deposited directly onto the WC-Co disc and the AlCrSiN top layer with the stoichiometry Al_25.5_Cr_21_Si_3.5_N_50_. The coating deposition was performed in a flowing nitrogen + argon (ratio N_2_/Ar = 6.67) atmosphere under a working pressure that was constantly maintained at 4 Pa. The cathode currents were I*_AlSi_* = 110 A and I*_Cr_* = 70 A. The coating technique, where two cylindrical and rotating cathodes were used, can be seen in Figure 1.

During the deposition, negative bias voltages of −80 V, −100 V, and −120 V were applied to the substrates. The selection of the bias voltage interval can be explained by the fact that these values belonged to the most often used values of bias voltage in the industry, as well as the substrate temperature, which was kept at 470 °C. The coating thickness was 4 ± 0.10 µm. A summary of the deposition parameters is listed in Table 1 [38].

### 2.2. Measurement of the Coating Thickness, Nanohardness, and Coating-Substrate Adhesion

Nanohardness measurements were performed on as-deposited coatings using the Anton Paar NHT^2^ nanoindentation device, using the Oliver & Pharr method with the Berkowich diamond indentor. The loading rate was 300 mN/min with a dwell time of 3 s and an unloading rate of 300 mN/min. The loading force was linear during the whole measurement. The thickness of the coating was measured by Calotest. Calotest is designed to quickly characterize coating thickness. The simple method offerred a fast and accurate means of checking the thickness of any kind of coating, whether a single or multilayered stack [39]. The method included the measurement of the characteristic parameters of the crater developed due to the wear of the sample surface caused by the steel ball with diamond emulsion [40]. The values were used for determination of the indentation depth, which was set on 10% of the coating thickness for each sample. Therefore, the substrate effect on the overall hardness can be neglected. The 20–25 indents were performed for each sample, and the average hardness H (GPa) and Young Modulus E_it_ (GPa) with standard deviation (GPa) were obtained [38].

The static Mercedes indentation test was carried out on a Rockwell-type hardness tester. Indentation tests were performed with a diamond cone under the load of 1500 N. The indents were studied by the scanning electron microscope (SEM). The coating-substrate adhesion was classified at six levels, where the first four were acceptable grades (HF1–4; only a small amount of coating delamination is allowed), and the two other were unacceptable grades (HF5 and HF6; a large area of the coating delamination occurs) [41,42].

### 2.3. Evaluation of the Preliminary Experiment

#### 2.3.1. Nanohardness

**Figure 2:**
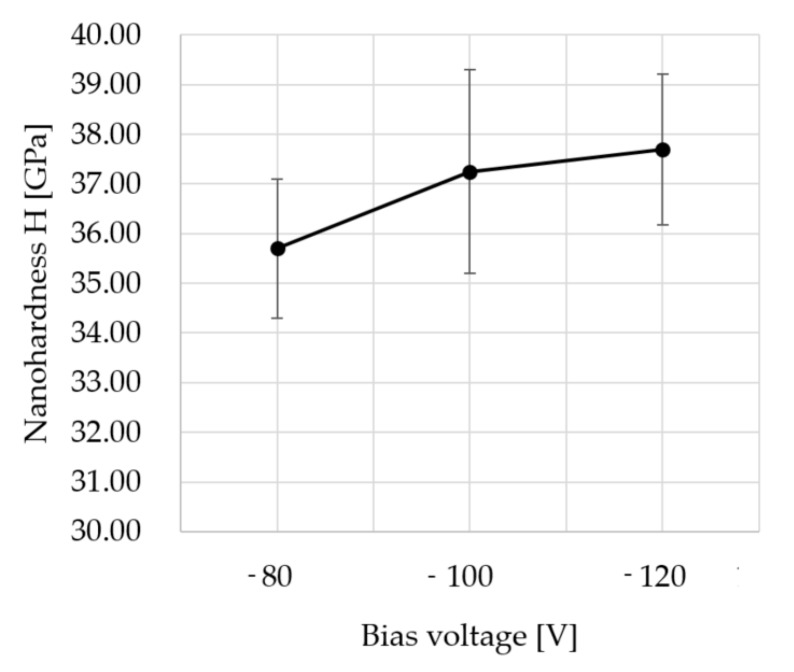
Nanohardness H (GPa) as a function of the bias voltage (V) [38].

Measurements of nanohardness were performed to determine and choose the most promising coating for real cutting conditions. Figure 2 shows the nanohardness of the AlCrSiN coatings as a function of the bias voltage. From the graph, it is clear that if bias voltage increased, nanohardness increased from −80V to −120V. The maximum nanohardness of as-deposited coatings was 37.7 ± 1.5 GPa for samples obtained at −120 V [38].

#### 2.3.2. Coating-Substrate Adhesion

**Figure 3:**
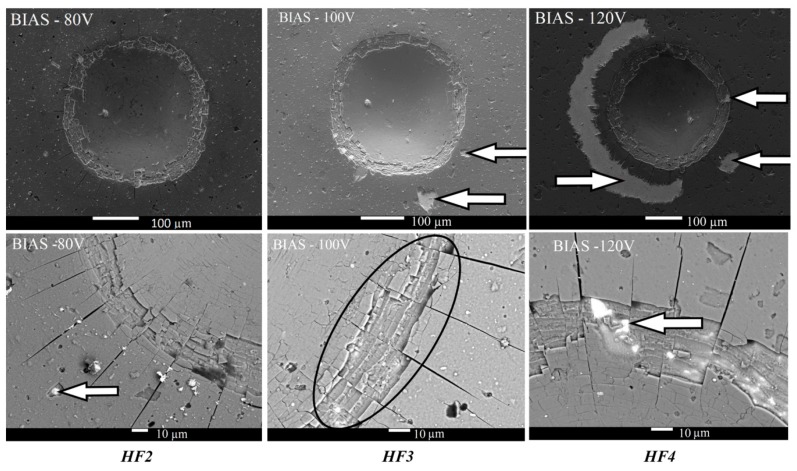
Scanning electron microscope (SEM) images of the Rockwell-C indents and evaluation of coating-substrate adhesion depending on the different bias voltage [38].

Coatings deposited at lower bias voltages showed very good adhesion HF2 (−80 V) (left column in Figure 3) and good adhesion HF3 (−100 V) (middle column in Figure 3). The increase in the bias voltage (−120 V) resulted in the deterioration of the adhesion strength. However, coating-substrate adhesion was still acceptable (HF4). At low bias voltages (≤−100 V), typical features were radial microcracks and cohesion failure, such as the area designated by the black ellipse (middle column in the bottom line in Figure 3). In this area, the coating-to-coating separation occurred on the inside of the indent. Cohesion failure was observed ar each indentation mark, as can be seen in detail in the lower line of Figure 3. The white arrows show areas where coating delamination was observed. In the case of the highest bias voltage (−120 V), cohesive failure persisted in the indent, whereas an adhesion failure (delamination of the coating from the substrate) started to occur on the edges of the indent (right column in Figure 3). Therefore, evaluation of coating-substrate adhesion for indentation marks of bias voltages −120V would be disputable due to one large area (white arrow on the left side) and one small area (white arrow on the right side), where the coating was peeled. These areas of coating delamination were observed in a distance of about 30 µm ÷ 70 µm from edges of the indent, but only small coating delamination between two microcracks was found on the edges of the indent (top white arrow on the right side). For this case, if coating delamination was not observed on the edge of indent but appearred some distance from the edge of indent, then coating-substrate adhesion was classified as acceptable [38].

## 3. Materials and Methods

In the experiment, the influence of cutting edge radius *r_n_* sizes on the tool life during the turning of austenitic stainless steel material was investigated. Moreover, the impact of surface roughness and time of edge preparation with surface finishing on the coating-substrate adhesion was identified. The other aim was to compare the various methods of edge preparation with surface finishing, with respect to the tool life of the coated cutting tools. For this purpose, the cemented carbide turning inserts were used as samples where the edge preparation with surface finishing was applied. The cemented carbide turning inserts with a designation of CNMG 120408E-SM had the same chemical composition as the previous cemented carbide discs in the preliminary experiment: 89.6% of WC, 10% of Co, and 0.4% of other carbides. The dimensions of the cemented carbide turning insert were 12.9 mm × 12.9 mm × 4.76 mm. This type of substrate was selected concerning the PVD coating process, workpiece material, and semi-finishing strategy. In the experiment, the required sizes of cutting edge radii were established to be in a range from 20 μm to 60 μm. These values of cutting edge radius size present a wide range of cutting strategies where the machined material must also be taken into account.

### 3.1. Edge Preparation with Surface Finishing

With the support of the Dormer Pramet tool Company, two industrially-used methods of edge preparation with surface finishing were applied to the samples. Brushing is a method that uses rotary brushes with abrasive SiC grains. Cemented carbide turning inserts were prepared by brushing in order to improve the surface roughness, with respect to the cutting edge radius sizes. Wet microblasting is a method that uses the forcibly propelling stream of Al_2_O_3_ abrasive medium against a surface of cemented carbide cutting insert under high pressure. Cemented carbide turning inserts were prepared by wet microblasting to smooth a surface roughness and prepare the required cutting edge radii.

**Figure 4:**
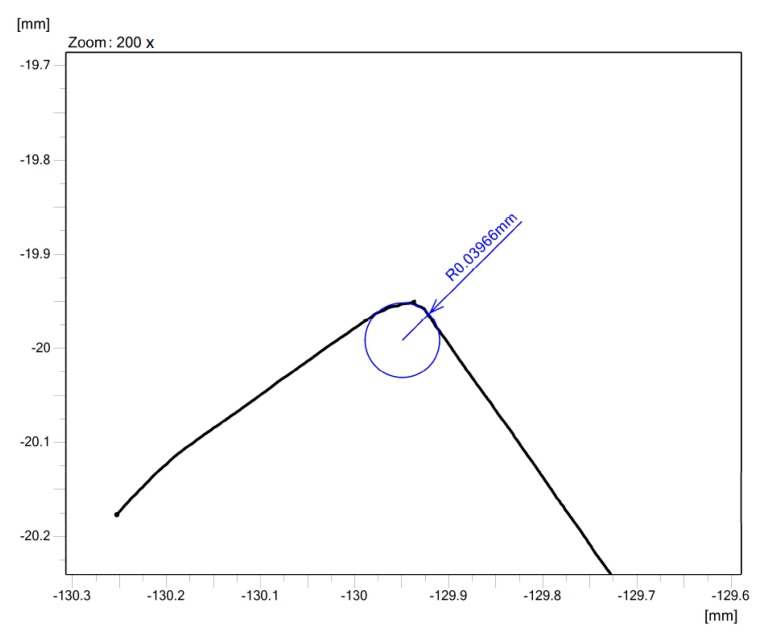
Measurement of the cutting edge radius size.

The time of edge preparation with surface finishing *t* was set to achieve the required cutting edge radius (*r_n_*) sizes. The cemented carbide turning inserts were prepared with the three cutting edge radius sizes: *r_n_* = 20 μm, *r_n_* = 40 μm, and *r_n_* = 60 μm. Each value of the cutting edge radius corresponds to the time of surface finishing. MarSurf XCR 20 roughness and the contour measuring station and Accretech Contourecord measuring device were used to measure the cutting edge radius sizes. An example of measurement of cutting edge radius size is shown in Figure 4. After the process of edge preparation with surface finishing, the cemented carbide turning inserts were cleaned in the ultrasonic unit. The surface roughness parameters, *Ra* and *Rz*, were then measured on the flank of cemented carbide, turning inserts for testing the coating-substrate adhesion on this flank surface.

### 3.2. Selected Deposition Parameters of AlCrSiN Coating for Cemented Carbide Turning Inserts

The results of the preliminary experiment showed that the maximum nanohardness of as-deposited coatings was 37.7 GPa for samples obtained at −120 V. Furthermore, the evaluated coating-substrate adhesion was also acceptable. Therefore, the following deposition parameters were used to deposit the cemented carbide turning inserts for the experiment, as seen in Table 2. After cleaning, all cemented carbide turning inserts were deposited by the selected AlCrSiN nanocomposite hard coating when deposition conditions were kept constant.

### 3.3. Measurement of the Coating-Substrate Adhesion

The static Mercedes indentation test was performed on a Rockwell-type hardness tester. Indentation tests were carried out with a diamond cone under the load of 1500 N intended for thin coatings. The coating-substrate adhesion was observed on SEM and classified from HF1 (excellent adhesion) to HF6 (poor adhesion), according to the amount of cracking or coating delamination around the edges of the indent.

### 3.4. Tool Life Cutting Test

**Figure 5:**
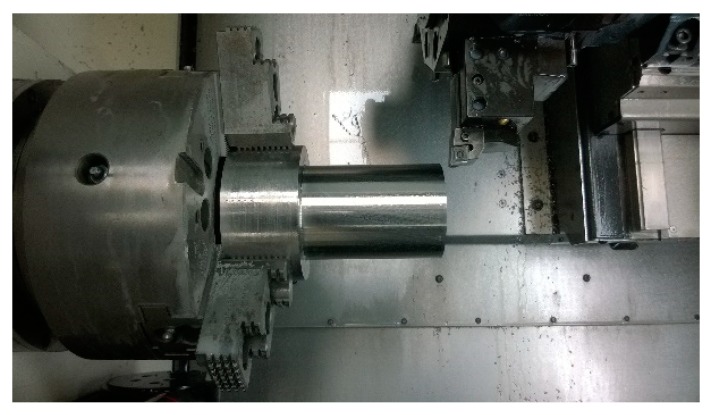
Workplace of the turning center during the tool life cutting test.

For tool life cutting tests, the DMG CTX alpha 500 turning center was used (Figure 5). The CNMG 120408E-SM cemented carbide turning insert and PCLNL 2020K12 toolholder were selected for this research. The selected workpiece material was austenitic stainless steel material of DIN EN X6CrNiTi18-10 (AISI 321) grade in this experiment. Chemical composition is shown in Table 3. The round bar with dimensions of ø 100 × 150 mm was used in the tool life cutting test.

In the tool life cutting test, the influence of cutting edge radius sizes on the tool life during turning of austenitic stainless steel material was investigated. Cemented carbide turning inserts with the 4 μm thick AlCrSiN coatings used in a tool life cutting test had the following three cutting edge radius sizes:

- *r_n_* = 24 μm
- *r_n_* = 44 μm
- *r_n_* = 64 μm

The large notch as a part of the width of flank wear was formed on the cutting edge. It was a dominant tool wear type because a too large notch caused the breakage of the cemented carbide insert. Therefore, the width of flank wear was measured during the cutting in a particular period of time. The flank wear criterion was set to be 0.24 mm of the width of flank wear. The oil-in-water emulsion with 4% oil concentration was used as a cutting fluid. The cemented carbide turning inserts with a specific size of cutting edge radius were tested three times.

The cutting parameters that mostly affect the chip forming during the machining process are the feed and depth of cut. The minimum and maximum values of the feed and depth of cut are related to the size and shape of the chip former. If values of the depth of cut and feed were smaller during the machining process, the chip did not break and chip forming was not problematic. The cutting speed was studied experimentally. Research of Vopat et al. [38] discusses the investigation of cutting parameters for the cutting tool life test in detail. The cutting parameters of AlCrSiN-coated cemented carbide turning inserts for the tool life cutting test are given in Table 4.

## 4. Results and Discussion

### 4.1. Edge Preparation with Surface Finishing

**Figure 6:**
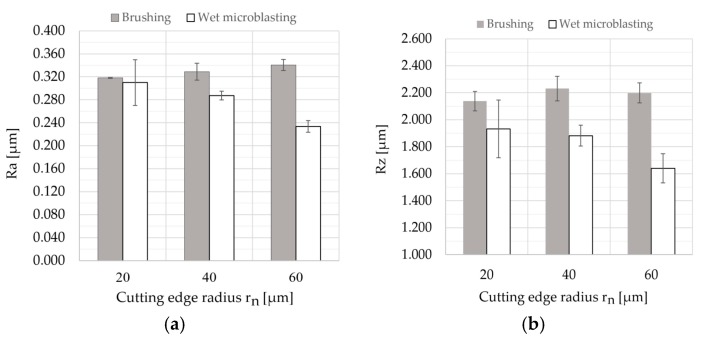
Measured values of surface roughness parameters of cemented carbide turning inserts prepared by brushing and wet microblasting: (**a**) Measured values of *Ra* (arithmetical mean deviation of the assessed profile); (**b**) measured values of *Rz* (maximum height of the profile).

The average values of surface roughness parameters, *Ra* and *Rz*, are depicted in Figure 6. Since all surface roughness parameters were measured five times, error bars were added.

Each value of the cutting edge radius *r_n_* corresponds to the time of edge preparation with surface finishing *t* (Table 5). It can be seen from the results that the surface roughness parameters were directly proportional to the time of brushing. On the other hand, an increase in the time of wet microblasting led to a decrease in the surface roughness parameters.

### 4.2. Coating-Substrate Adhesion

**Figure 7:**
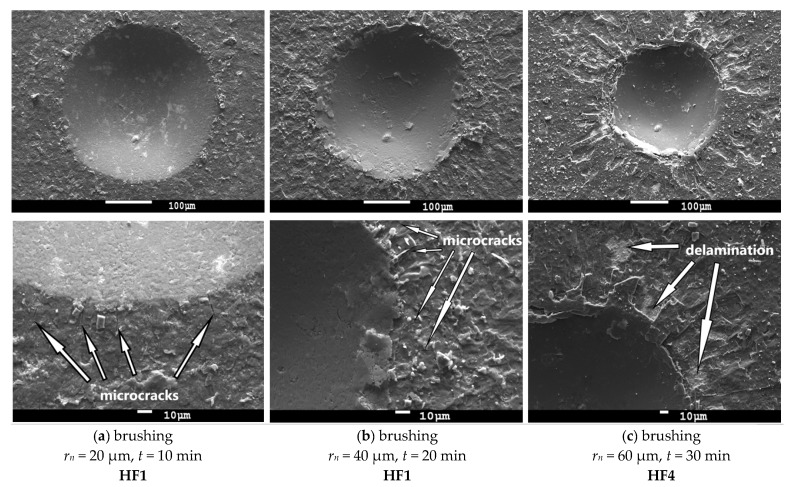
SEM images of the Rockwell-C indents and evaluation of coating-substrate adhesion on cemented carbide turning inserts prepared by brushing.

**Figure 8:**
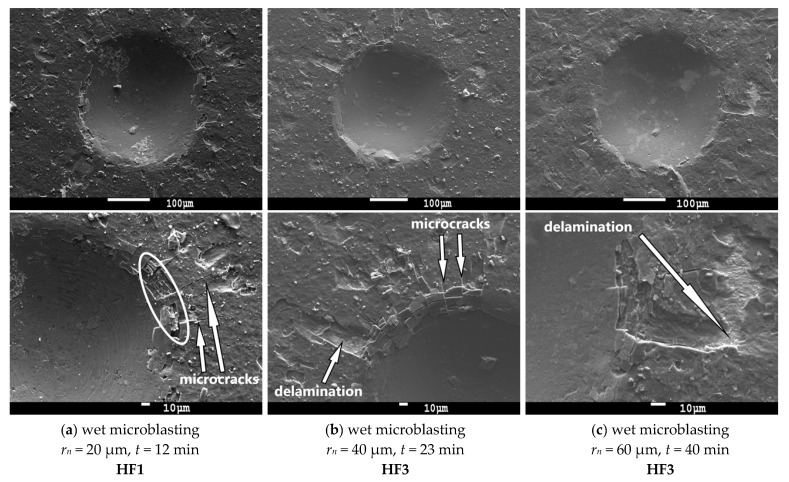
SEM images of the Rockwell-C indents and evaluation of coating-substrate adhesion on cemented carbide turning inserts prepared by wet microblasting.

Figure 7 and Figure 8 showed indents after the static Mercedes indentation test on cemented carbide turning inserts. According to our observations, the coating-substrate adhesion was acceptable in the case of all cemented carbide turning inserts. However, the results of the static Mercedes indentation test showed that the adhesion strength of the coating to the substrate was affected by the used edge preparation method with surface finishing and its time. As can be seen from SEM images, higher time of edge preparation with finishing decreased coating adhesion to the cemented carbide turning inserts.

The coatings deposited on cemented carbide turning inserts, prepared by brushing (Figure 7) for 10 min (left column) and 20 min (middle column), showed a very good adhesion (HF1). The typical features were radial microcracks on the edges of the indent. Coating delamination was observed on the cemented carbide turning insert, prepared by 30 min brushing (right column). The white arrows show particular areas where the coatings were delaminated. The coating-substrate adhesion was still acceptable (HF4).

In the case of cemented carbide turning inserts prepared by wet microblasting (Figure 8) for 12 min, typical features, such as cohesion failure in the white border and radial microcracks, can be seen in Figure 8 (left column). The coating-substrate adhesion was classified as very good (HF1) because no coating delamination was observed. The coatings deposited on cemented carbide turning inserts, prepared by brushing for 23 min (middle column) and 40 min (right column), demonstrated a good adhesion (HF3). The only small areas of coating delamination were observed between two microcracks on the edges of the indent.

It is obvious from the results that the long time of edge preparation with surface finishing resulted in decreasing coating-substrate adhesion. It can be caused by the removal of cemented carbide from the substrate by abrasive treatment.

**Figure 9:**
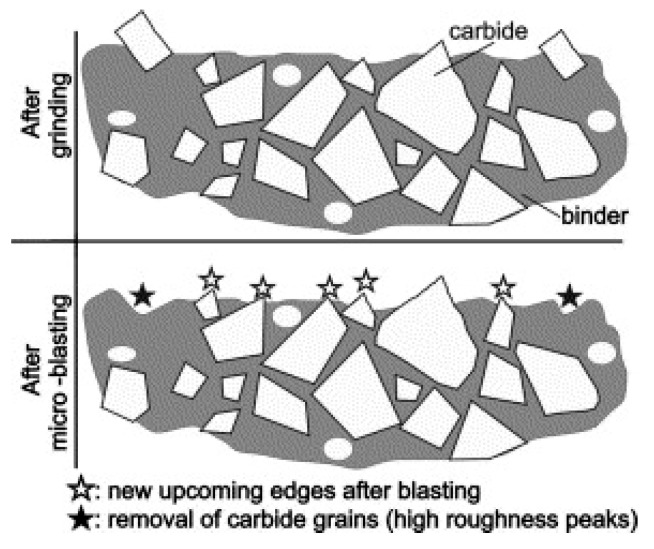
Scheme of surface layer of cemented carbide substrate before and after microblasting treatment [43].

The principle of removal of cemented carbide by microblasting treatment is described in research of Bouzakis et al. [43]. The method of microblasting is often used on cemented carbide inserts to reduce large peaks on the surface and decrease the surface roughness parameters. Since cobalt is more ductile in comparison to WC grains, cobalt is removed from the surface to be coated during the edge preparation with surface finishing. Large roughness peaks were mainly reduced due to the removal of carbide grains, and new smaller peaks were revealed. This observation (Figure 9) was investigated by Bouzakis et al. [43].

The same principle of removal of cemented carbide by another treatment that used abrasive particles for preparation can be used. The surface roughness parameters significantly increased or decreased with respect to previous surface roughness of the finished surface. However, the increasing or decreasing of surface roughness values can be slower after a particular time of brushing. The brushing treatment used the rotary brushes with abrasive SiC grains.

In brushing, the material removal took place by impact of abrasive SiC particles on the edge, flank, and rake face, and it formed a more irregular cutting pattern. Thus, higher values of *Rz* can be expected after brushing [44].

From the results, differences among the measured values of *Ra* and *Rz* were too small, and, therefore, it can be stated that the time of brushing had no significant effect on surface roughness parameters, *Ra* and *Rz*, in range of 10 to 30 min.

According to Bouzakis et al. [43], small roughness peaks contribute to the enhancement of coating-substrate adhesion due to the better mechanical binding of the deposited coating on the substrate surface.

However, it is very important to emphasize that surface roughness of the substrate has limiting values where coating-substrate adhesion is improved. Therefore, a too smooth surface of the substrate can causes a decrease in the coating-substrate adhesion [45]. This can be explained by the following: The surface with larger surface roughness parameters can be better deposited on the substrate than smooth parameters due to the larger total contact area of the coating and substrate [44].

On the other hand, a too rough surface of the substrate can also cause a decrease in the coating-substrate adhesion. This can be observed when the value of *Ra* is larger than 1 μm [45,46,47].

The main reason for this problem was the increase of local stresses in roughness points and the increase in defects in the interface due to the effect of shading [47].

The results of the Mercedes indentation test showed that coating-substrate adhesion slightly decreased with increasing values of surface roughness parameters, in case of wet-microblasting, but the difference was very small. In the case of brushing, the values of the surface roughness parameter *Rz* that were measured after 10 min of brushing (*Rz* = 2.138 μm) and after 30 min of brushing (*Rz* = 2.199 μm) were approximately the same, while the coating-substrate adhesion decreased from HF1 to HF4. It is obvious from the results that such a small variance of values of surface roughness parameters had no significant effect on the coating-substrate adhesion in this low range. However, it is clear from the results that the long-time of edge preparation with surface finishing caused the negative changes on the surface of the substrate, resulting in a decrease of coating-substrate adhesion. Since cobalt is more ductile in comparison to WC grains, cobalt was removed from the surface to be coated during the edge preparation with surface finishing [43], as mentioned above (Figure 9). The long process of the edge preparation with surface finishing caused the effect of cobalt leaching. The percentage of cobalt was decreased with an increase in the time of edge preparation with surface finishing on the top of the surface of the substrate. This fact could be the reason for the decrease in the coating-substrate adhesion.

### 4.3. Evaluation of the Tool Life Cutting Test

**Figure 10:**
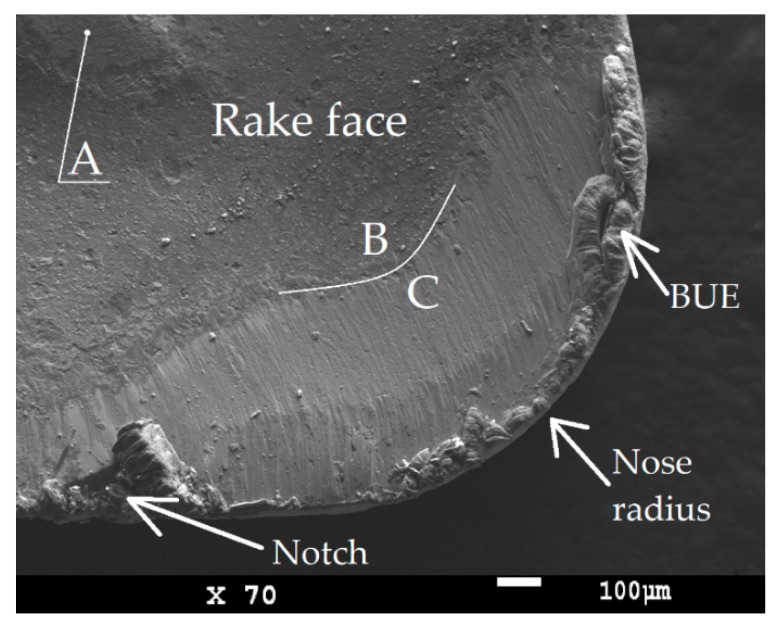
SEM image of worn cemented carbide turning inserts (on rake face) used in the experiment.

The SEM image (Figure 10) shows a worn sample (coated cemented carbide turning insert), where a typical tool wear type during machining tough material is illustrated. It demonstrates a higher strain hardening tendency. Tool wear on the rake face (Crater wear), as seen from the Figure 10 area C, is usually the result of an abrasion process caused by interaction between the chip and cemented carbide turning insert. The dividing line demonstrates the area where the coating remained intact (area B) and the area where the chips have worn out the coating (area C). EDS (Energy Dispersive X-Ray Spectroscopy) analysis of these areas was used to examine the chemical composition of the top layer. It was also observed that, in area (A) on the chip former, the coating was worn out by the chip. This tool wear type was not significant enough to negatively affect the chip forming.

**Figure 11:**
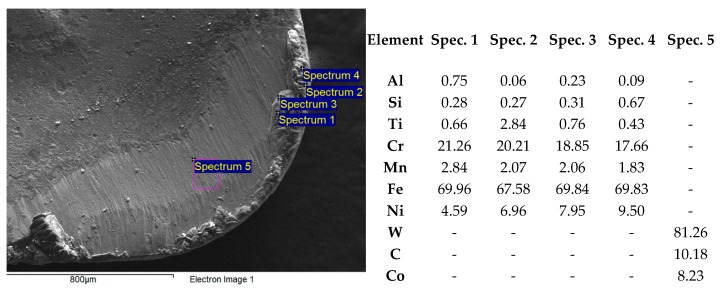
SEM image of the area of the built-up-edge (BUE) and worn rake face (area C) and EDS (Energy Dispersive X-Ray Spectroscopy) elemental analysis of selected regions.

The built-up-edge (BUE) was probably the result of tool and workpiece affinity associated with temperature and its respective cutting speed. The particular elements from the workpiece material were investigated by EDS analysis (Figure 11). It is complicated to avoid BUE during the machining of austenitic stainless steel material because increasing the cutting speed could lead to a rapid decrease in the tool life. However, this type of tool wear was not dominant because the built-up edge did not break off the cutting edge or cemented carbide turning inserts in the experiment, as can be seen in Figure 10.

Suggestions based on SEM observations were confirmed by the analysis of chemical composition (Figure 11). Areas presented on the cutting edge (Spectrum 1–4) were rich in Cr, Ni, Mn, and Fe, which are related to the chemical composition of the workpiece material. Only W, C, and Co elements were detected on the rake face (area C), since the coating was worn out during the cutting process.

**Figure 12:**
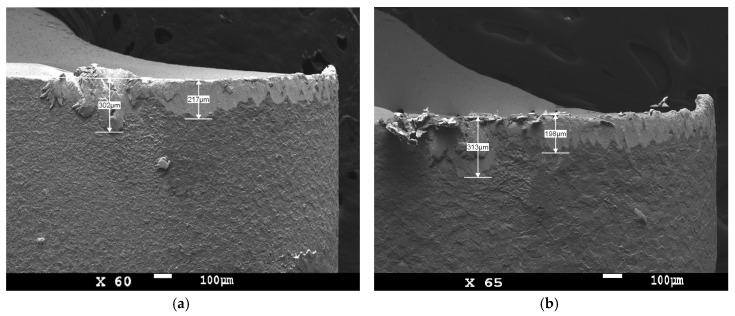
SEM images of flank wear with a large notch on the flank of worn cemented carbide turning insert prepared by (**a**) brushing; (**b**) wet microblasting.

As seen from SEM images (Figure 12), the large notch (>300 μm) as a part of the width of flank wear was observed on the cutting edge. It was a dominant tool wear type because a too large notch caused the breakage of the cemented carbide turning insert.

**Figure 13:**
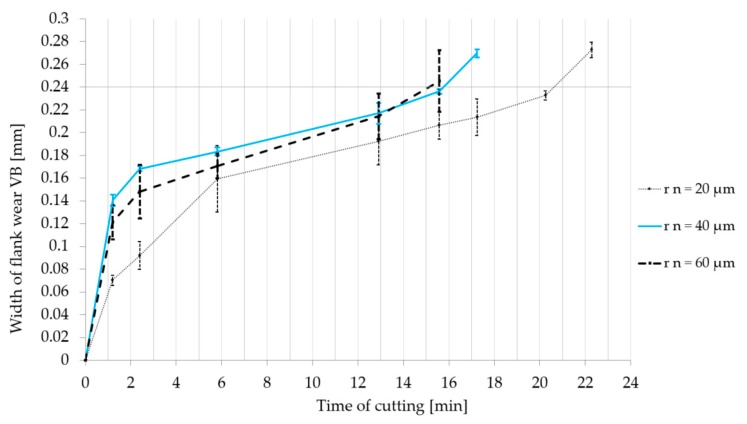
Dependence of the width of flank wear value VB on the time of cutting. Cemented carbide turning inserts were prepared by brushing.

**Figure 14:**
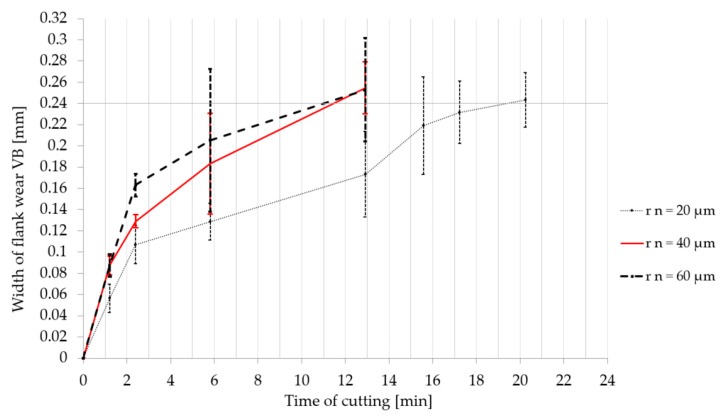
Dependence of the width of flank wear value VB on the time of cutting. Cemented carbide turning inserts were prepared by wet microblasting.

The graphs in Figure 13 and Figure 14 express the evolution of the width of flank wear (VB) during the cutting in a particular period of time. Every type of cemented carbide turning insert was tested three times in order to exclude the impact of other factors involved in the process, as mentioned before. Therefore error bars and the average value of the width of flank wear was calculated and inserted to the graph. The results show that cemented carbide turning inserts with a larger cutting edge radius were worn out faster during the machining.

**Figure 15:**
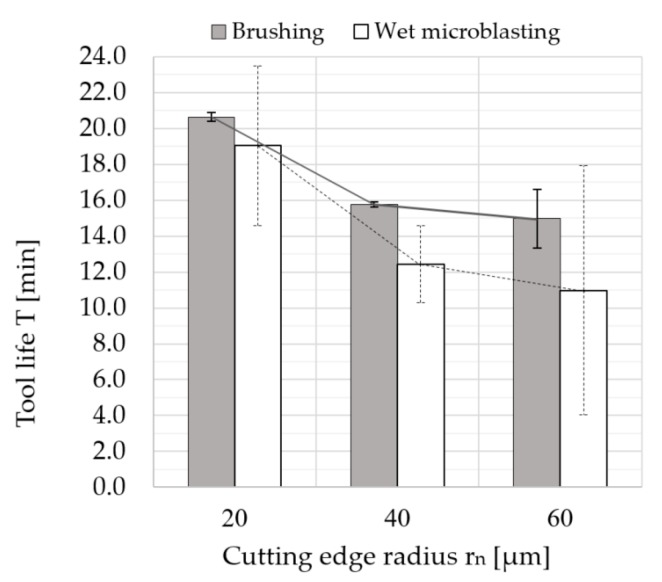
Tool life T (min) as a function of the cutting edge radius size r_n_ (μm).

In Figure 15, the graph shows the dependence of the tool life on the cutting edge radius size. The graph demonstrates the average values of particular tool lives. The tool life of coated cemented carbide turning inserts prepared by brushing with a cutting edge radius of about *r_n_* = 24 μm was approximately 30% longer than that of the cemented carbide turning inserts with *r_n_* = 44 μm. The tool life of the coated cemented carbide turning inserts prepared by brushing with *r_n_* = 24 μm was approximately 36% longer than that of the coated cemented carbide turning inserts with *r_n_* = 64 μm. Meanwhile, the tool life of the coated cemented carbide turning inserts prepared by wet microblasting with *r_n_* = 24 μm was about 70% higher than that of the cemented carbide turning inserts with a cutting edge radius of 44 μm. Moreover, the coated cemented carbide turning inserts prepared by wet microblasting with *r_n_* = 24 μm showed approximately 77% longer tool life compared to the coated cemented carbide turning inserts with *r_n_* = 64 μm.

The notch was formed on the cutting edge as a progressive increase in the flank wear. The notch can be observed during the machining the hardened material or during the machining the material that was hardened by plastic deformation [48,49,50,51,52,53].

**Figure 16:**
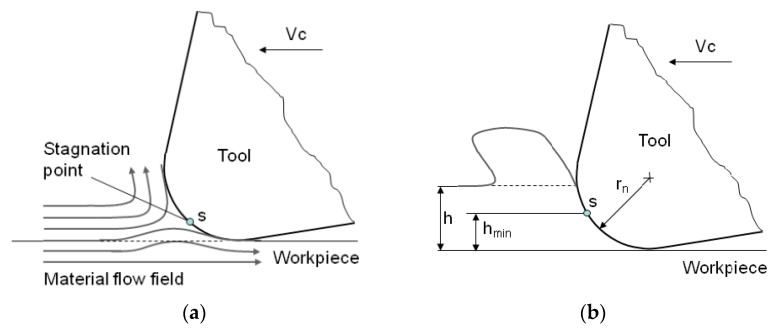
Deformation zone (**a**) concept of stagnation point [56]; (**b**) minimum cutting thickness [28].

These results were investigated during the machining of austenitic stainless steel material, which shows a higher strain hardening tendency. From the graph (Figure 15), the results show that tool life increased with a decrease in the size of the cutting edge radius. This can be caused by the strain hardening that is related to the stagnation point (Figure 16). Above this separation point S, material is assumed to flow into the chip. Below the stagnation point, it is considered to flow the workpiece to form the machined surface [54,55,56]. The position of the stagnation point can be affected by a lot of factors. Rodriguez [28] proposed the model of the relationship among minimum cutting thickness and friction angle and minimum and cutting edge radius. If the cutting edge radius was larger, the minimum cutting thickness was larger because the position of stagnation point was moved higher on the basis of this model. Under the stagnation point, the workpiece material was pressed and therefore the machined surface was significantly harder than the work surface. For this reason, the larger cutting edge radius made greater strain hardening, which resulted in a harder machined surface.

## 5. Conclusions

The article examined the influence of edge preparation with surface finishing on the tool life of AlCrSiN-coated carbide cutting tools and coating-substrate adhesion. The preliminary experiment aimed to determine the optimal coating deposition parameters for the cutting tools made of cemented carbide. The impact of deposition parameters on the coating-substrate adhesion and nanohardness was investigated. The results from the preliminary experiment set the parameters of the coating deposition for the tool life cutting test. The static Mercedes indentation test was performed on a Rockwell-type hardness tester to determine coating-substrate adhesion on cemented carbide samples. Nanocomposite AlCrSiN hard coating had a very good coating-substrate adhesion at the lowset bias voltage of −80 V (HF2). The adhesion strength of the coating to the substrate decreased with an increase of the bias voltage, but it was still acceptable. At the highest bias voltage of −120 V, the adhesion was classified as acceptable. The appropriate deposition conditions were determined for coating the cemented carbide turning inserts.

In the experiment, the impact of the surface roughness and time of edge preparation with surface finishing on the coating-substrate adhesion was identified. The cemented carbide turning inserts (89.6% of WC, 10% of Co, and 0.4% of other carbides) with edge preparation were used as substrates. Two methods of edge preparation with surface finishing (brushing and wet microblasting) were applied to the cemented carbide turning inserts to form the cutting edge radius. The time of brushing had no significant effect on surface roughness parameters, *Ra* and *Rz*, in ranges of 10 to 30 min, because the differences among the measured values of *Ra* and *Rz* were too small. This can be caused by the removal of cemented carbide from the substrate by abrasive treatment, as wasdescribed.

The results of the Mercedes indentation test showed that the long time of edge preparation with surface finishing resulted in a decrease of coating-substrate adhesion. The reasons for decreasing the coating-substrate adhesion were discussed.

In the tool life cutting test, the influence of cutting edge radius sizes on the tool life during turning of the austenitic stainless steel material was studied. The AlCrSiN-coated samples were prepared with three cutting edge radius sizes: *r_n_* = 24 μm, *r_n_* = 44 μm, and *r_n_* = 64 μm. The results showed that cemented carbide turning inserts with a larger cutting edge radius were worn out faster during the machining. This can be caused by the strain hardening that is related to the stagnation point. The larger cutting edge radius made greater strain hardening, which resulted in a harder machined surface.

In further research, authors will focus on the influence of cutting edge radius sizes on strain hardening of machined surfaces during machining of austenitic stainless steel material.

## Figures and Tables

**Table 1:** Summary of deposition parameters for AlCrSiN coating in the preliminary experiment [38].

I_AlSi_ (A)	I_Cr_ (A)	Bias (V)	Temperature (°C)	Pressure (Pa)	N_2_ (sccm)	Ar (sccm)
110	70	80–120	470	4	200	30

**Table 2:** Selected deposition parameters for AlCrSiN coating in the experiment.

I_AlSi_ (A)	I_Cr_ (A)	Bias (V)	Temperature (°C)	Pressure (Pa)	N_2_ (sccm)	Ar (sccm)
110	70	120	470	4	200	30

**Table 3:** Chemical composition of machined material DIN EN X6CrNiTi18-10 (AISI 321) grade.

Element	C	Si	Mn	P	S	Cu	Cr	Ni	Mo	Ti
wt. %	0.64	0.83	1.44	0.026	0.025	0.66	17.5	9.89	0.46	0.47

**Table 4:** Cutting parameters used in the tool life cutting test.

Cutting Speed *v_c_* (m/min)	Feed *f* (mm)	Depth of Cut *a_p_* (mm)
127	0.2	1

**Table 5:** Time of edge preparation with surface finishing.

Cutting Edge Radius	Time *t* of Brushing	Time *t* of Wet Microblasting
*r_n_* = 20 μm	10 min	12 min
*r_n_* = 40 μm	20 min	23 min
*r_n_* = 60 μm	30 min	40 min
